# Disclosure of cancer diagnosis and prognosis: a survey of the general public's attitudes toward doctors and family holding discretionary powers

**DOI:** 10.1186/1472-6939-5-7

**Published:** 2004-12-01

**Authors:** Hiroaki Miyata, Hisateru Tachimori, Miyako Takahashi, Tami Saito, Ichiro Kai

**Affiliations:** 1 Department of Social Gerontology, School of Health Sciences and Nursing, Graduate School of Medicine, University of Tokyo, Japan; 2 National Institute of Mental Health, National Center of Neurology and Psychiatry, Japan

## Abstract

**Background:**

This study aimed to ask a sample of the general population about their preferences regarding doctors holding discretionary powers in relation to disclosing cancer diagnosis and prognosis.

**Methods:**

The researchers mailed 443 questionnaires to registered voters in a ward of Tokyo which had a socio-demographic profile similar to greater Tokyo's average and received 246 responses (response rate 55.5%). We describe and analysed respondents' attitudes toward doctors and family members holding discretionary powers in relation to cancer diagnoses disclose.

**Results:**

Amongst respondents who wanted full disclosure about the diagnosis without delay, 117 (69.6 %) respondents agreed to follow the doctor's discretion, whilst 111 (66.1 %) respondents agreed to follow the family member's decision. For respondents who preferred to have the diagnosis and prognosis withheld, 59 (26.5 %) agreed to follow the doctor's decision, and 79 (35.3 %) of respondents agreed with following family member's wishes.

**Conclusions:**

The greater proportion of respondents wants or permits disclosure of cancer diagnosis and prognosis. In patients who reveal negative attitudes toward being given a cancer disclosure directly, alternative options exist such as telling the family ahead of the patient or having a discussion of the cancer diagnosis with the patient together with the family. It is recommended that health professionals become more aware about the need to provide patients with their cancer diagnosis and prognosis in a variety of ways.

## Background

Cancer ranks as the third leading cause of death worldwide, accounting for approximately 12 % of all recorded deaths [1]. As cancer is sometimes fatal and its treatment often involves invasive medical procedures and medication, it has a great impact on patients' lives. The extent to which physicians should inform patients of their diagnosis and prognosis poses a difficult decision in clinical settings. Previous studies show that a patient's cancer diagnosis is not routinely disclosed in many cultures in Africa [2], Eastern and Southern Europe [3-6], and the Middle East [7]. Even in the United States, where most doctors follow informed consent guidelines which includes informing patients of their diagnosis as standard clinical practice, problems still exist regarding the accurate provision of prognosis information [8].

In Japan, historically, physicians have withheld discussing cancer diagnoses directly with patients [9]. However, since the early 1990s, due to the increased understanding and adoption of informed consent policy and practice, physicians have gradually begun to inform patients of their cancer diagnosis in clinical practice [10,11]. In many cases, however, details regarding prognosis are still concealed from patients, especially if the condition is incurable [12,13]. While some physicians provide full information from the outset, others provide no information at all, even withholding basic diagnosis information [14]. The National Cancer Centre (The core national institution for developing cancer treatment, research and policy) has compiled a set of guidelines for cancer disclosure. However, each hospital has deferring policy and practice [9]. No law or regulation stipulates that doctors are required to obtain informed consent from patients. Given this context, there are demonstrated needs to develop concrete guidelines and to promote cancer disclosure based on patients' preferences.

In Japan, the patient, family and doctor are the main players in cancer disclosure. According to legal precedents in Japan, doctors are given a wide range of discretionary powers regarding disclosure [14-16]. As a rationale for holding discretionary power, doctors report a number of compelling reasons such as the need to protect patients from psychological distress caused by disclosure of the diagnosis, families' wishes for non-disclosure to patients, and the fact that most patients themselves do not wish to be told the truth [9,17,18]. However, several case-control studies report that there is no relationship between cancer disclosure and mental harm [19-21]. As family members are more reluctant than patients to disclose the truth [11,22], patients' needs for information are often unsatisfied in Japan where physicians often discuss the cancer diagnosis with family prior to informing the patient [23,24]. Doctors' discretionary powers and families' powers of attorney need to be reconsidered in the light of patients' preferences. This study's aim was to ask the general population whether they, in the event of developing cancer, preferred doctors' (or family members') discretionary powers regarding disclosure of the cancer diagnosis and prognosis.

## Methods

This study was a cross-sectional, stratified random sampling survey of the general population in their 40s to 50s. As people over 60 years old are epidemiologically more at risk of having cancer, we excluded them not only because it seemed harmful to ask about these experiences, but also because there was a possibility that their responses would be affected by their experiences. Participants were selected from eligible voters in 'A' ward in the Tokyo Metropolitan Area. We chose 'A' ward as a representative area of Tokyo because various social indices such as the proportion of the elderly population, average length of education, and population growth rate were consistent with the Tokyo average [25]. The researchers mailed 443 questionnaires in October 2002 and received 246 responses (response rate 55.5 %). Amongst the respondents, 26 (10.5%) people had been diagnosed with cancer sometime in the past. As there were no significant differences in the responses of those who had been diagnosed with cancer and those who had not, we included these 26 respondents in the analysis. There were also no significant differences between those who were relatives of a cancer patient or were not and those who had dealt with cancer in their role as medical staff or had not. The sample size was determined by the need to provide adequate numbers to be able to detect differences among disclosure preferences with some degree of statistical certainty.

The questionnaire was developed in consultation with 6 medical staff and 19 patients. The questionnaire presented a hypothetical scenario in which "The doctor discovers terminal cancer, but the patient does not know yet." to each respondent, and asked about preferences regarding diagnosis and prognosis disclosure; "How would you want to be told, if you were in such a situation". Answer choices for disclosure preferences regarding diagnosis were: 1."I would not want to be given any information regarding my diagnosis [non-disclosure]", 2. "I would like to obtain information regarding my diagnosis of a general nature but not in detail" 3." "I would like to be given all information regarding my diagnosis [full-disclosure]". Choices for disclosure on the prospects of complete recovery (CR) and expected length of survival (LS) were: 1. "I would not want to be given any information about the prospects of CR and LS [non-disclosure]", 2." I would like to obtain information on the prospects of CR and LS of a general nature but not in detail. [partial-disclosure]", 3. "I would like to be told about my prospects of CR and LS eventually. However, I would like to receive only general information on the prospects of CR (LS) when I am initially informed about the disease [postponed full-disclosure]", and 4. "I would like to be told about my prospects of CR and LS without delay. [immediate full-disclosure]". The reason for providing s answers allowing partial-disclosure was based on research by Akabayashi[26] which indicated that many Japanese were accustomed to and commonly preferred ambiguous or graded answers rather than polarised ones.

Respondents were asked about their attitudes toward doctors and family members holding discretionary powers regarding cancer diagnosis disclosure. In order to compare the attitudes and characteristics of respondents who preferred immediate diagnosis and prognosis and those who did not, analysis was carried out twice. In the first analysis we included respondents who did not choose "full diagnosis and prognosis without delay", and we included the data from the remaining respondents that explained the reason for allowing to receive immediate diagnosis and prognosis. In the second analysis we included respondents who did want to receive diagnosis and prognosis, and we included data from the rest of the respondents about their reasons for preferring the withholding of diagnosis and prognosis. We also asked about preferences regarding the cancer disclosure process, such as whether people would like to obtain information ahead of their family.

The questionnaire also included the trait part of the Japanese version of the State-Trait Anxiety Inventory (STAI), which assesses the personality predisposition to anxiety [27-29]. The Japanese version of STAI is a widely used and standardized test. In the present sample, the trait part of STAI for Cronbach's α = 0.90.

Firstly, we calculated all respondents' disclosure preferences regarding diagnosis, CR and LS. Secondly, we calculated the attitudes toward doctors and family members holding prognosis discretion of respondents who preferred to be given diagnosis information directly, and those who did not. Wilcoxon's test was used to examine the differences between the attitudes held toward doctors and family members holding discretionary powers between these two groups. Statistical analyses were conducted using SPSS Version 11.5J.

## Results

The socio-demographic characteristics of the respondents are shown in Table 1. The mean age of the 246 respondents was 49.8 years (± 6.2 years). More than half (N = 143: 58.1 %) were female, 78 (31.7 %) had graduated from college, and 32 (13.0 %) were living alone.

**Table 1:** Characteristics of the respondents. (N = 246)

	Mean	SD
Age (yr)	49.8	6.2
STAI (total score)	41.4	9.9

	N	%

Sex (female)	143	58.1 %
College graduates	78	31.7 %
Living alone	32	13.0 %
Married	186	75.6 %
Living with adult child	85	26.1 %
Living with infant child	98	39.8 %
Principal household earner	133	54.1 %
Non-religious	185	75.2 %

Respondents' preferences regarding diagnosis and prognosis disclosure are shown in Table 2. Regarding diagnosis, 85.4 % of respondents wanted full-disclosure, 11.3 % wanted partial disclosure and 2.9 % wanted non-disclosure. In the case of the prospect of a complete recovery; 35.7 % of respondents wanted an immediate full-disclosure, 17.2 % wanted a postponed full-disclosure, 39.2 % wanted partial-disclosure, and 2.9 % wanted no disclosure. Regarding the expected length of survival; 32.2 % of respondents wanted an immediate full-disclosure, 11.4 % wanted a postponed full-disclosure, 50.0 % wanted partial-disclosure, and 6.4 % wanted no disclosure.

**Table 2:** Disclosure preferences regarding diagnosis and prognosis

	Non-disclosure	Partial-disclosure	Full-disclosure	
Diagnosis (N = 239)	7 (2.9 %)	27 (11.3 %)	204 (85.4%)	

	Non-disclosure	Partial-disclosure	Postponed Full-disclosure	Immediate Full-disclosure

Prospect of Complete recovery (N = 238)	7 (2.9 %)	105 (39.2 %)	41 (17.2 %)	85 (35.7 %)
Expected Length of Survival (N = 236)	15 (6.4 %)	118 (50.0 %)	27 (11.4 %)	76 (32.2 %)

**Figure 1:**
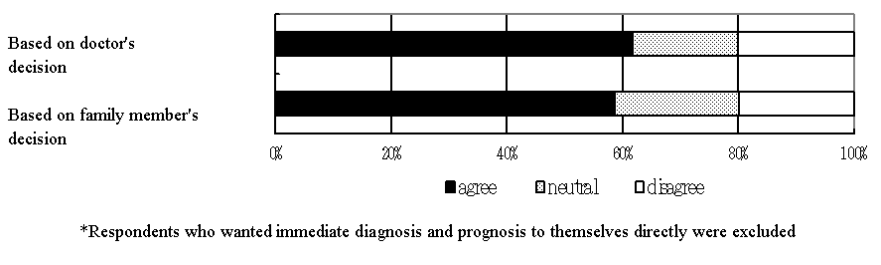
Preference for who should decide whether to give immediate diagnosis and prognosis. N = 175.

**Figure 2:**
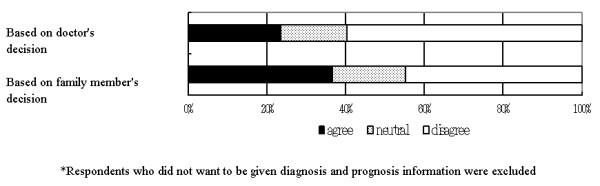
Preferences for who should decide whether to withhold diagnosis and prognosis. N = 240.

Regarding the contextual reason for wanting to receive full diagnosis and prognosis information without delay, 117 (69.6 %) respondents agreed to follow the doctor's initiative and 111 (66.1 %) of the respondents agreed to follow the with family member's decision [Figure 1]. The Wilcoxon test found no significant difference between these two groups (z = 0.186, p = 0.853). As for the reason for wanting the diagnosis and prognosis information to be withheld, 59 (26.5 %) of the respondents agreed to follow the doctor's initiative, and 79 (35.3 %) of respondents agreed to follow family member's wishes [Figure 2]. Wilcoxon test found significant differences between these two groups (z = 6.470, p < 0.001).

Regarding the cancer disclosure process, more than half the respondents (N = 136; 55.3 %) answered that they would like to obtain diagnosis and prognosis information ahead of their family, a third (N = 82: 32.3 %) answering that would like to receive information with their family together at the same time. Only 26 (32.3%) respondents preferred to obtain this information after the doctor had already informed their family.

## Discussion

Regarding preferences relating to diagnosis and prognosis, only 2.7 % of the respondents wanted no information regarding a cancer diagnosis. In addition to considering to tell or not to tell, the extent to which physicians should inform patient of diagnosis and prognosis poses a difficult decision in clinical settings. However, more than two-thirds (68.7 %) wanted full diagnostic and general prognostic information in a general nature but not in detail or wanted to be told about their prognosis eventually. Less than a third (28.9 %) wanted full information regarding diagnosis and prognosis without delay. These results suggest that a disclosure policy which provides patients with full information on diagnosis and general information on prognosis can satisfy the majority of patients' preferences. The results also suggest that any disclosure policy should also try to acknowledge and meet patients' wishes of being informed together with their families, and of being given information at a later time.

Nevertheless, some patients do not want any information regarding their cancer diagnosis. In the clinical setting, medical staff needs to develop policy and procedures that can deal with the needs of patients who do not want any information as well as those patients and who want complete information immediately. The priority in identifying these types of patients over-rides other factors which affect patients preferences regarding diagnosis and prognosis such as patient characteristics and seriousness of cancer (previous research conducted by the authors [30]). Regarding those respondents who did not want to be given a diagnosis directly, those who preferred to follow a family member's decision were significantly larger than those who would prefer a doctor to decide. If patients reveal negative attitudes toward being given a cancer diagnosis at the time of initial consultation and testing, it may still be effective to tell the patient's family ahead of the patient or to have a discussion of cancer disclosure together with the family.

Despite the data that indicates a mix of patients' preferences regarding cancer diagnosis, it may not be necessary for doctors to make choices regarding diagnosis by actually knowing individual patients' preferences. As opposed to those who would prefer no information, a greater proportion of respondents wanted to receive full information, even contrary to their preferences. Two other surveys with the general public show a similar tendency of patients wanting more information regarding cancer diagnosis than they used to. Asahi Newspaper found that regarding one's own cancer diagnosis and prognosis, in 1989, 59% of respondents wanted disclosure, which increased to 76%) in 2000 [31]. Similarly, Yomiuri Newspaper found that in 1994, 70% of respondents preferred being given information about a cancer diagnosis that increased to 78% in 2001 [32]. Thus the importance of providing information is widely supported by the majority of the general community.

To simulate the fact that cancer results in a variety of disease outcomes for patients, we used scenarios with a range of severities in the outcomes of the cancer. As a result, there is little difference between respondents who had experienced cancer disclosure as a patient and those who did not, and the diagnosis preferences revealed in this study (full-disclosure, 85.4%) are consistent with previous studies (Seo [18], 85.7%: Miura [33], 88.1%). These findings suggest that this study's method succeeded in simulating a situation that reflected some degree of reality for respondents who had been given a cancer diagnosis in the past.

This study has several limitations. Although the response rate to this study was moderate for a general population survey, we acknowledge that the characteristics of the respondents might not be wholly representative of the general population. Also, because we restricted participants to adult inhabitants in an urban area in Japan, further research is required to test the validity of these findings.

It is recommended that health professionals become more aware about the need to provide patients with options to be given their cancer diagnosis and prognosis in a variety of ways. The greater proportion of respondents wants or permits disclosure of cancer diagnosis and prognosis. However, in patients who reveal negative attitudes toward being given a cancer disclosure directly, alternative options should be made available such as telling the family ahead of the patient or having a discussion of the cancer diagnosis with the patient together with the family. Further research with people aged over-sixty is needed to test the applicability of these findings to older age groups.

## Competing interests

Although partial funding for this study was provided by the Education Ministry within the Japanese government, the views and opinions expressed in this report are those of the authors and not those of the funding organisation.

## Authors' contributions

HM planned and conducted the survey, carried out the analysis, and wrote this paper.

HT, MT, TS and IK made close supervision and extensive support. All authors read and approved the final manuscript.

## Pre-publication history

The pre-publication history for this paper can be accessed here:


